# Polymorphic segmental duplication in the nematode *Caenorhabditis elegans*

**DOI:** 10.1186/1471-2164-10-329

**Published:** 2009-07-21

**Authors:** Ismael A Vergara, Allan K Mah, Jim C Huang, Maja Tarailo-Graovac, Robert C Johnsen, David L Baillie, Nansheng Chen

**Affiliations:** 1 Department of Molecular Biology and Biochemistry, Simon Fraser University, Burnaby, British Columbia, V5A 1S6, Canada

## Abstract

**Background:**

The nematode *Caenorhabditis elegans *was the first multicellular organism to have its genome fully sequenced. Over the last 10 years since the original publication in 1998, the *C. elegans *genome has been scrutinized and the last gaps were filled in November 2002, which present a unique opportunity for examining genome-wide segmental duplications.

**Results:**

Here, we performed analysis of the *C. elegans *genome in search for segmental duplications using a new tool–OrthoCluster–we have recently developed. We detected 3,484 duplicated segments–duplicons–ranging in size from 234 bp to 108 Kb. The largest pair of duplicons, 108 kb in length located on the left arm of *Chromosome V*, was further characterized. They are nearly identical at the DNA level (99.7% identity) and each duplicon contains 26 putative protein coding genes. Genotyping of 76 wild-type strains obtained from different labs in the *C. elegans *community revealed that not all strains contain this duplication. In fact, only 29 strains carry this large segmental duplication, suggesting a very recent duplication event in the *C. elegans *genome.

**Conclusion:**

This report represents the first demonstration that the *C. elegans *laboratory wild-type N2 strains has acquired large-scale differences.

## Background

Genomes are highly dynamic. Comparative genome analysis has revealed extensive differences, including inversions, transpositions, reciprocal translocations and duplications, among genomes of different species, as well as among genomes of different strains within the same species. In particular, duplications had been observed and studied long before any genome sequencing projects were initiated. For example, the *Bar *"gene" duplication in the fruit fly *Drosophila melanogaster*, which was found to be important in determining eye size, was identified cytologically in the 1920s [1]. Now, with the availability of genome sequences of many species, a large number of studies have been carried out to detect *in silico *and in a genome-wide manner the presence of such duplications [2]. Duplications can be classified into three types based on their scales: whole genome duplications, segmental duplications, and single gene duplications. In 1970s, Susumu Ohno proposed that gene duplication is the driving force for the generation of new genes and novel biochemical processes [3].

*Caenorhabditis elegans *is a widely used model organism for its small size, short life cycle, well-defined development, ease of manipulation, as well as a compact genome. In *C. elegans*, gene duplications have been found to be responsible for the formation of many gene families, including chemosensory gene families [4-10], transcription factors [11], ABC transporters [12,13], and gene families important in host-pathogen interactions [14]. In contrast to the extensive analyses of individual gene duplications in *C. elegans*, large scale segmental duplications have received little attention, although they are known to exist [15-17].

In this project, we have carried out a genome-wide analysis of segmental duplications in *C. elegans *using a new program called OrthoCluster [18], and we have experimentally assessed the polymorphism of the largest pair of duplicons in different wild-type (N2) strains of *C. elegans *as well as the wild isolate–Hawaiian strain (CB4856). Given that we ran OrthoCluster at the gene level, in which each chromosome is represented as a set of genes with their corresponding order and strandedness, the term "segmental duplication" is used here to describe any group of one or more genes that are found duplicated in the genome.

## Results

### Identification of genome-wide segmental duplications

We applied OrthoCluster to identify genome-wide segmental duplications in *C. elegans*. OrthoCluster can identify "perfect segmental duplications"–duplications containing no mismatches, "imperfect segmental duplications"–duplications containing a certain level of mismatches (genetic interruptions), as well as synteny blocks among multiple genomes [18]. In this report, we call each duplicated segment of genes a duplicon [19].

#### Perfect segmental duplications

We identified 1,980 perfect segmental duplications, which generate 3,484 duplicons [see Additional file 1]. Note that the number of duplicons is not exactly twice the number of segmental duplications because the same regions can be duplicated more than once. The majority of the segmental duplications (1,364, or 68.9%) are intrachromosomal and can be further categorized as tandem (567/1,980, or 28.6%) when the corresponding duplicons are found adjacently, or as dispersed (797/1,980, or 40.3%) when at least one gene is separating them.

**Figure 1:**
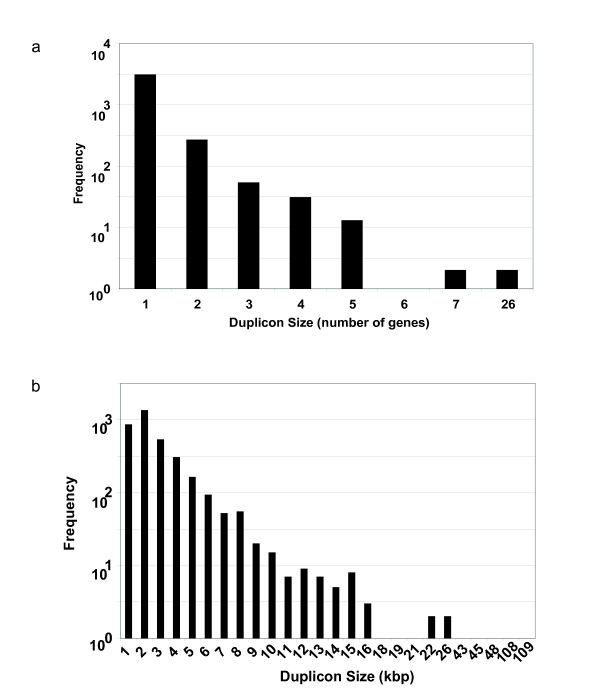
**Size distribution of perfect duplicons in *C. elegans *genome**. (a) Size distribution of all perfect duplicons in the *C. elegans *genome measured in number of genes. (b) Size distribution of all perfect duplicons in the *C. elegans *genome measured in kb. Each N value in the x-axis represent all those duplicons that fall in the range [N-1..N) kb. The y-axis represents the frequency in a logarithmic scale (base 10) of the frequency of a specific duplicon size. Thus, those bins with no visible bar mean that only one duplicon is observed for that particular value.

Sizes of the identified duplicons vary dramatically, ranging from one to 26 genes (Figure 1a), or from 234 bp to 108 Kb in size (Figure 1b). The majority of these duplicons contain single genes (3,112, or 89.3%), while a few contain more than ten genes, consistent with previous observations [16] [see Additional file 2]. The duplicons are not evenly distributed in different chromosomes, with *Chromosome V *having significantly more duplications than other chromosomes (p < 0.01, Fisher's Exact test). The largest pair of duplicons is located on the left arm of *Chromosome V*, and each duplicon contains 26 genes with a genomic span of 108 Kb. Although the presence of this large segmental duplication has been reported in previous studies [15-17], detailed analysis has not been pursued.

#### Imperfect segmental duplications

Search for imperfect segmental duplications revealed larger duplicons, suggesting that some smaller neighboring perfect duplicons can merge to form larger imperfect ones. As a result, the number of duplicons identified decreased from 3,484 (for perfect segmental duplications) to 3,447, generated by 1,955 imperfect segmental duplications [see Additional file 3].

### Molecular comparison of the two largest duplicons

**Figure 2:**
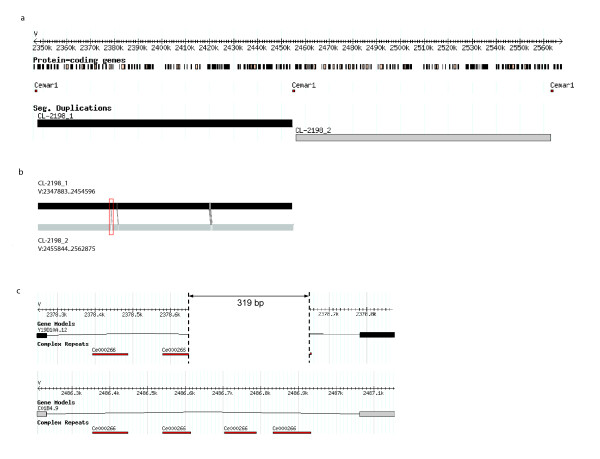
**The two largest duplicons in the *C. elegans *genome**. (a) Genome browser image of the largest duplicons CL-2198_1 (depicted in black) and CL-2198_2 (depicted in gray), and flanking *Cemar1 *transposons (shown in red). (b) Alignment of the two largest duplicons indicate the locations of the small differences. From 5' to 3': (1) 319 bp deletion in first duplicon. (2) A single nucleotide insertion ('C') in first duplicon at 2,381,150 bp. (3) A single nucleotide difference ('T" is the first duplicon at 2,420,123 bp and 'C' in the second duplicon at 2,528,402 bp) (4) A single nucleotide difference ('A' in the first duplicon at 2,420,126 bp and 'T' in the second duplicon at 2,528,405 bp). (5) A single nucleotide difference ('T' in the first duplicon at 2,420,132 bp and 'C' in second duplicon at 2,528,411 bp). (6) A triplet difference ('TAC' in the first duplicon from 2,420,134 bp to 2,420,136 bp and 'ACT' in the second duplicon from 2,528,413 bp to 2,528,415 bp). (c) The 319 bp unique sequence in the largest duplicon. Multiple copies of Ce000266 repetitive element are located in the region. The upper and lower panels show the upstream and downstream copies of the largest duplicons, respectively.

To further characterize the largest segmental duplication, we compared the two duplicons generated by the largest segmental duplication at the base pair level. First, we observed that the two duplicons are found in tandem on *Chromosome V *between 2,346 Kb and 2,565 Kb in the canonical *C. elegans *genome sequence that is hosted at WormBase http://www.wormbase.org[20]. Each duplicon contains 26 putative protein-coding genes, most of which are putative chemosensory genes based on WormBase (WS180) curation. Additionally, we found identical copies of mariner-like transposable element *Cemar1 *[21,22] flanking the duplicons (Figure 2a). Multiple sequence alignment of the DNA sequences of these transposons indicated that they are nearly identical (99.4% identity). In contrast, the regions upstream of the beginning of the first *Cemar1 *and downstream of the third *Cemar1 *(Figure 2a) show no significant similarity. Next, we compared DNA sequences of the two duplicons, and found that they have 99.7% sequence identity, with only six small differences (Figure 2b). Considering the large size of these duplicons (106,714 bp and 107,032 bp), such high level of similarity is rather surprising. The biggest difference is a 319 bp deletion found in the upstream duplicon (Figure 2c). Other differences are limited to one to three nucleotides, and notably, all differences are located in either intergenic regions or within introns of current gene models (Figure 2b).

Given that these two duplicons are virtually identical, we expect all 26 gene models contained in each of these duplicons to be identical. To our surprise, based on the current WormBase (WS180) annotation, only 13 of the 26 pairs are identical (Table 1) [see Additional file 4], suggesting that many of these gene models are defective, and thus need to be improved. We have thus attempted to improve these gene models using existing EST sequence data and their similarity to known paralogous genes that are curated by WormBase curators (see methods). All updated gene models have been submitted to WormBase [see Additional file 5].

**Table 1:** List of genes within the duplicated region.

Sequence Name	EST Support	Paralog Sequence Name	EST Support	Identical?	Method of repair
Y19D10A.7	NS	F56A4.9	NS	N	Longest: F56A4.9
Y19D10A.9	PS	F56A4.2	PS	Y	N.A.
Y19D10A.8	NS	F56A4.10	NS	N	Longest: F56A4.10
Y19D10A.6	NS	F56A4.1	NS	N	Evidence: nas-2
Y19D10A.10	NS	F56A4.11	NS	N	Longest: F56A4.11
Y19D10A.11	NS	F56A4.12	NS	N	Longest: Y19D10A.11
Y19D10A.12	PS	C01B4.9	PS	N	Longest: C01B4.9
Y19D10A.5	FS	C01B4.8	FS	Y	N.A.
Y19D10A.4	PS	C01B4.7	PS	Y	N.A.
Y19D10A.16	FS	C01B4.6	FS	Y	N.A.
Y19D10A.15	NS	C01B4.5	NS	Y	N.A.
Y19D10A.2	NS	C01B4.3	NS	Y	N.A.
Y19D10A.13	NS	C01B4.10	NS	Y	N.A.
Y19D10A.1	NS	C01B4.1	NS	N	Evidence: str-257
Y19D10A.17	NS	Y45G12C.8	NS	Y	N.A.
C13B7.3	NS	Y45G12C.7	NS	Y	N.A.
C13B7.6	PS	Y45G12C.16	PS	N	Longest: Y45G12C.16
C13B7.4	NS	Y45G12C.9	NS	Y	N.A.
C13B7.5	NS	Y45G12C.10	NS	N	Evidence: str-119
C13B7.2	NS	Y45G12C.6	NS	N	Evidence: str-120
C13B7.1	NS	Y45G12C.5	NS	Y	N.A.
F56A4.5	NS	Y45G12C.4	NS	N	GeneWise: E02C12.11
F56A4.6	NS	Y45G12C.11	NS	N	Longest: F56A4.6
F56A4.4	PS	Y45G12C.3	PS	Y	N.A.
F56A4.7	NS	Y45G12C.12	NS	Y	N.A.
F56A4.3	FS	Y45G12C.2	FS	N	*

Each gene pair is shown in order of appearance from 5' to 3'. The "Method of repair" column suggests a way to fix those gene models that have different peptide sequence, given the lack of supporting information for better improvement. Longest: suggests taking the longest peptide sequence as the correct model. Evidence: suggests considering as correct the member of the pair that has been reported as "person evidence" in WormBase. GeneWise: suggest a paralog gene that can be used to predict a gene structure in the region within each member of the pair. Each gene is characterized in terms of EST data support as NS (Not Supported) if no intron is supported, PS (Partially Supported) if at least one intron is not supported, and FS (Fully Supported) if all introns are supported by EST data. *: see text.

### Experimental characterization of the largest duplicons in *C. elegans*

**Figure 3:**
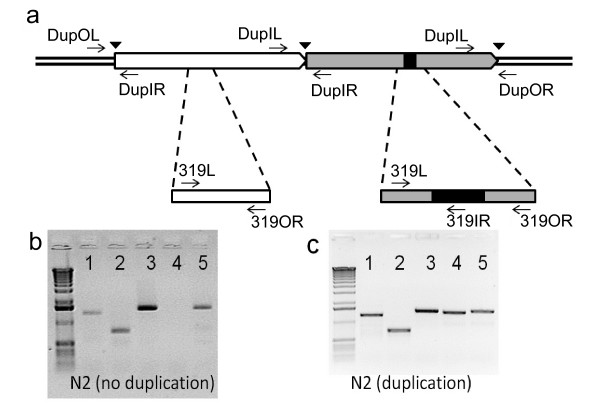
**PCR analysis of the largest tandem segmental duplicons**. (a) A schematic illustration of the largest duplicons, with PCR primers used for genotyping labeled. (b) A representative gel for strains that do not carry the largest duplication. (c) A representative gel for strains carrying the largest duplication. Lane 1 shows PCR product using primers 319L and 319OR; lane 2 shows PCR product using primers 319L and 319IR; lane 3 shows PCR product using primers DupOL and DupIR; lane 4 shows PCR product using primers DupIL and DupIR; and lane 5 shows PCR product using primers DupIL and DupOR.

The high level of similarity between these two largest duplicons in the *C. elegans *genome prompted us to hypothesize that they were generated very recently and thus not all wild-type (N2) strains carry them. To test this hypothesis, we genotyped 76 of the N2 strains, received from the researchers in the *C. elegans *community, for the presence of these duplicons. For each strain, we examined (1) the presence of the junction between the two largest duplicons (Figure 3a, lane 4) and (2) the presence of the 319 bp unique sequence (Figure 3a, lanes 1 and 2). Results showed that the 76 samples processed can be divided into two groups: a group of 47 samples that don't carry the largest duplicon pair (Figure 3b), and a group of 29 samples that carry the largest duplicon pair (Figure 3c). In addition, this tandem duplication was not found in the *C. elegans *CB4856 strain, an isolate from a Hawaiian island. Thus, we conclude from these results that the N2 worms, which all originated from a common ancestor first established in Sydney Brenner's lab in 1960s [23,24], had become polymorphic in this genomic region in laboratory.

This conclusion is further supported by two interesting patterns that emerged from our genotyping assays. First, 16 of the 17 CGC (*Caenorhabditis *Genetics Center) strains (obtained from different *C. elegans *labs) don't have the largest duplicons. This includes the strain from Donald Riddle, who originally set up the CGC. The only one "CGC N2 strain" (among these 17 CGC strains) that carries the largest duplicons is thus likely not a real CGC but was in fact obtained from an alternative source. Second, all 11 strains that were obtained from Robert Horvitz's lab and from the labs that obtained their N2 strain directly or indirectly from the Horvitz lab (according to senders) contain the largest duplicons. We have also tested the existence of the junction in the cosmid F56A4, which was created and used in the *C. elegans *genome sequencing project [15]. PCR results clearly showed the presence of the duplication junction in the cosmid F56A4 (data not shown), suggesting that this pair of duplicons also exist in the *C. elegans *strain used for the *C. elegans *genome sequencing project. Together, these observations support our hypothesis that this large tandem duplication arose as a result of a recent event, after the N2 strain was established as a laboratory strain in the early 1970s.

### Tandem segmental duplications and transposons

The presence of nearly identical *Cemar1 *transposons flanking the largest duplicons suggests a possible role of these transposons in the duplication event (Figure 2a). The fact that these duplicons are found in tandem and in a head-to-tail orientation, together with the close to 100% transposon DNA identity suggests that this segmental duplication occurred by an unequal crossing over event facilitated by the presence of the *Cemar1 *transposons. The expected outcome of an unequal recombination event is two types of chromosomes: one with the duplicated region and one with a deletion of the same region. Unlike duplication, deletion of 26 genes could lead to a reduced evolutionary fitness and loss of the strain.

In order to examine whether this mechanism accounts for other observed tandem duplications, we selected all duplications in the *C. elegans *genome that are larger than 1,000 bp that show more than 90% identity at the DNA level and examined their correlation to the distribution of transposable elements. Altogether 31 pairs of tandem duplicons (Table 2), including the largest tandem duplicons described above, were examined and only five were found to be associated with neighboring transposons, suggesting that transposable elements may play a role in the formation of some, but not all, tandem segmental duplications. This association is not significantly different from random (*p *= 0.56). In addition, for all cases associated with transposons, except the largest duplicons, transposons are found in the neighborhood of one duplicon but not perfectly flanked by transposons at edges. Alternatively, it is possible that most of the transposable elements have moved away from the tandem duplication regions after the duplication event.

**Table 2:** Tandem segmental duplications in *C. elegans *of size 1,000 or larger

Coordinates Dup1	Coordinates Dup2	Matches (bp)	Orientation	N Genes Dup1	N Genes Dup2	Associated Transposons
V:2347883..2454596	V:2455844..2562875	106707	F	26	26	Cemar1
V:8813143..8850811	V:8855237..8892906	37642	F	11	13	TC5, Cer9
III:1251054..1258404	III:1259414..1266845	7339	F	4	4	NO
IV:12471444..12478970	IV:12478981..12486507	7527	F	3	3	NO
X:226651..231363	X:236067..240779	4713	F	3	3	NDNAX1
IV:5241391..5244977	IV:5246223..5249809	3587	R	3	3	NO
I:12627236..12632544	I:12635161..12640469	5304	R	2	2	NO
X:1940626..1945025	X:1949155..1953554	4399	F	2	2	NO
V:9087269..9088593	V:9089256..9090580	1325	R	2	2	NO
X:3558880..3563952	X:3567445..3572527	4985	F	1	1	NO
IV:13129621..13133199	IV:13135213..13138791	3579	F	1	1	NDNAX3
I:11616806..11620253	I:11623105..11626552	3448	R	1	1	NO
IV:4348439..4351841	IV:4352611..4356013	3403	R	1	1	NO
X:4333166..4336008	X:4339618..4342467	2823	R	1	1	NO
II:11757121..11759167	II:11759614..11761660	2047	R	1	1	NO
V:13967844..13969831	V:13974541..13976528	1988	R	1	1	NO
III:7171786..7173519	III:7174002..7175735	1734	R	1	1	NO
IV:16339625..16341334	IV:16342450..16344159	1710	R	1	1	NO
III:11787629..11789338	III:11790417..11792126	1709	R	1	1	NO
III:2433538..2435215	III:2436093..2437770	1678	R	1	1	NO
I:11303731..11305210	I:11308113..11309592	1480	R	1	1	NO
IV:1617460..1618943	IV:1622242..1623725	1481	R	1	1	NO
II:3588277..3589715	II:3592045..3593483	1439	R	1	1	NO
IV:9284870..9286382	IV:9292363..9293902	1466	F	1	1	NO
IV:2566235..2567558	IV:2569372..2570695	1324	R	1	1	NO
IV:16766557..16767821	IV:16768481..16769745	1265	R	1	1	LINE2
X:8319606..8320838	X:8322049..8323281	1233	R	1	1	NO
I:11355228..11356362	I:11358159..11359293	1135	R	1	1	NO
I:13890329..13891445	I:13893120..13894236	1117	R	1	1	NO
II:13079317..13080572	II:13082405..13083577	1173	R	1	1	NO
IV:5232834..5233864	IV:5236511..5237541	1031	R	1	1	NO

Interestingly, among all tandem duplications (Table 2), larger duplicon pairs (> 4,000 bp) tend to be arranged in a head-to-tail orientation (6 of 8, or 75%), while smaller ones are arranged in a tail-to-tail (inverted) orientation (3 of 23, or 13%, are in head-to-tail orientation within smaller duplicon pairs, p < 0.005, Fisher's Exact Test).

### Large tandem duplication polymorphism creates a new gene

**Figure 4:**
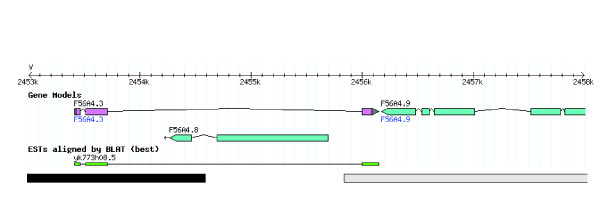
**Gene F56A4.3 at the junction of the largest pair of duplicons**. F56A4.3 gene model (shown in the "Gene Models" track) is fully supported by an EST sequence (shown in the "ESTs aligned by BLAT (best)" track). The black and grey bars represent the ends of the largest pair of duplicons.

A detailed examination of the junction between the two largest duplicons revealed that there is a gene (F56A4.3) flanking this junction that resides on both duplicons (Figure 4). This gene contains a glutathione S-transferase N-terminal domain and the gene model is fully supported by EST data. This EST data was generated by Yuji Kohara [25,26], who generated the EST library using a *C. elegans *strain that contains the largest segmental duplication, based on our genotyping result. Exons in F56A4.3 derived from exons in F15E11.10 (*srbc-15*) and Y45G12C.2 (*gst-10*). Thus, this large segmental duplication leads to the creation of a novel *C. elegans *gene through an exon shuffling mechanism [27,28]. The function of this putative new gene is being examined.

## Discussion and conclusion

In this project we applied OrthoCluster [18], our newly developed method for a gene-oriented detection and analysis of segmental duplications within the *C. elegans *genome. The versatility of this program allowed us to identify both perfect and imperfect segmental duplications, as well as to conclude that most of the identified duplicons are intrachromosomal and relatively small (Figure 1), consistent with previous observations [29-32].

The largest pair of duplicons that we identified is localized in tandem on *Chromosome V *and contains 26 genes. Our detailed analysis revealed that these duplicons are nearly identical, suggesting a very recent duplication event. This hypothesis is further supported by the following observations. First, these two duplicons are flanked by nearly identical *Cemar1 *transposons (Figure 2a), which may have caused a recent unequal crossing over event. Previous studies in *C. elegans *have shown that transposable elements can cause tandem duplications [33]. A recent study revealed that the *Cemar1 *transposons may be active in the *C. elegans *genome [34], which suggests that this segmental duplication was preceded by a transposition event of the *Cemar1 *element. Second, this large segmental duplication is strain-specific. Among 76 N2 strains genotyped, only 29 have the duplication. Since all of these 76 N2 strains were originated from a common ancestor strain, the large tandem segmental duplication might have occurred once after the establishment of N2 as a laboratory *C. elegans *wild-type strain in 1960s [23,35]. This ancestor strain was originally obtained from mushroom compost near Bristol, England, and was given to Sydney Brenner by Ellsworth Dougherty in the spring of 1964 [35]. From the Bristol strain, Sydney Brenner isolated a hermaphrodite and its progeny was used for establishing a line of hermaphrodites and a line of males. These were the founder stocks of the N2 strains [23]. Most likely, after the large segmental duplication was established, it was then propagated to other labs in the *C. elegans *community. This idea is consistent with the emerging patterns of the genotyping results–many duplication-carrying strains were obtained from labs that are related. Similarly, the strains that do not carry this large tandem duplication were obtained directly or indirectly from CGC. Additionally, the largest duplicon pair does not exist in the wild *C. elegans *isolate, the Hawaiian strain.

The expression and function of these 26 pairs of genes is largely unknown. Since many of these genes (16/52) are putative chemosensory genes, chemotaxis experiments [36] can be used to evaluated the impact of this duplication. The six differences could lie in regulatory elements and thus impact gene expression.

An unexpected result is that a new gene was created through exon shuffling as a byproduct of this large segmental duplication (Figure 4). The presence of this gene might be beneficial for the organism and thus helped to maintain these duplicons. The function of this new gene and its potential role in stabilizing the segmental duplication will be examined and reported separately.

An unsettled puzzle is the 319 bp unique sequence, which is found only in the downstream largest duplicon in the current *C. elegans *genome release (Figure 2c). In all 29 strains that carry the duplication, the 319 bp unique sequence is found in both duplicons. Interestingly, the sequence of the strain available at WormBase shows that this 319 bp sequence is found only in one of the duplicons–the downstream duplicon. Further examination of the genomic region harboring this putative deletion in the upstream duplicon shows that it is repetitive, containing several copies of a single complex repeat type (Ce000266) (Figure 2c). The difference between these two types of strains (the ones tested and the one used for the *C. elegans *sequencing project) could be explained by strain-specific deletion in the strain used for the *C. elegans *genome sequencing project. The possibility that the 319 bp sequence is an assembly error has not been ruled out.

Recent studies have proven that large genomic differences exist between the laboratory N2 *C. elegans *strain and the Hawaiian *C. elegans *strain, in addition to many SNPs discovered previously [37]. For example, Maydan and colleagues [38] discovered a ~2% gene variation between N2 *C. elegans *strain and the CB4856 Hawaiian *C. elegans *strain using array Comparative Genome Hybridization (aCGH) array. They uncovered significant variations, including deletions and copy-number differences. More recently, projects using Illumina Solexa sequencing methods revealed extensive differences (such as mutations and polymorphisms) even between different *C. elegans *laboratory strains [39,40] at the base-pair resolution. Our study reveals for the first time that different laboratory N2 strains can acquire and accumulate large-scale differences. Our discovery stresses the importance of taking into account variations in different laboratory strains when solving inconsistencies in results from different labs.

Our results, together with recent results using aCGH or Solexa sequencing methods, have thus clearly established that different N2 strains contain extensive differences in their genomic sequences. For robust research and for effective communication between different research groups, we recommend that labs should regularly start fresh from frozen *C. elegans *aliquot and should acquire N2 worms directly from CGC instead of from neighboring labs. More importantly, we recommend that each lab should keep a detailed record of the history of the N2 worms used. Furthermore, the N2 strain containing this large segmental duplication that is used in over one third of all *C. elegans *labs, should also be maintained and highlighted in CGC as a reference. Additionally, since the current *C. elegans *genome (hosted at WormBase) carries the largest duplicons (and potentially many additional differences) while the current CGC N2 strain does not, the CGC N2 strain, which is widely used in *C. elegans *labs, should be fully sequenced, assembled, analyzed, and compared with the current WormBase genome.

## Methods

### Genome-wide identification of segmental duplications using OrthoCluster

Genome sequences and annotation for *C. elegans *were obtained from WormBase [20] release WS180 http://ws180.wormbase.org/. Paralogs were determined by performing all-against-all blastp searches [41] with default parameters, with the exception of non-masking of low complexity regions, followed by filtering for an e-value less or equal than 1e^-40 ^and a percent identity of at least 70%.

For the detection of segmental duplications within the *C. elegans *genome, we have applied a newly developed program called OrthoCluster [18]http://genome.sfu.ca/projects/orthocluster/, by allowing no mismatches (for identifying "perfect segmental duplications") or a certain level of mismatches (for identifying "imperfect segmental duplications") within duplicons. OrthoCluster allows two types of mismatches: in-map mismatches, which correspond to genes that have paralogs in regions outside of the corresponding duplicon, and out-map mismatches, which correspond to genes with no paralogs in the *C. elegans *genome [18]. For the detection of perfect segmental duplications within the *C. elegans *genome, we allow no mismatches within duplicons, and preserve order and strandedness of the genes within the duplicons. For the detection of imperfect duplicons, order and strandedness were still required to be preserved, but a maximum of 15% of mismatched genes within duplicons were allowed.

### Sequence comparison between tandem duplicons

To identify differences between the largest duplicons and between transposons, alignments were carried out using ClustalW [42] with default parameters. Exact differences between the aligned copies were further obtained by systematically scanning through the alignments. For the tandem segmental duplications described in Table 2, we aligned each pair of duplicons (detected at the gene level using OrthoCluster) using ClustalW with default parameters, followed by trimming the edges that are not aligned to define the boundaries of the nearly-identical regions.

### Gene model improvement

In order to repair those gene models that were determined to be defective when comparing the two largest duplicons, the following set of rules was applied: (1) We first searched for EST sequences that supported the exon-intron boundaries as shown by the "EST aligned by BLAT (best)" and "EST aligned by BLAT (other)" tracks in WormBase; (2) If the gene model is not fully supported by ESTs, we then examined whether the gene model was curated by an expert; (3) if there is no evidence reported for the gene, we looked for their best curated paralogs, which are used as query to curate the defective gene model using GeneWise [43,44].

### Genome-wide detection of transposons and association with tandem segmental duplications

We obtained the Repbase 13.06 [45] library of repetitive elements for *C. elegans*, which contains all curated *C. elegans *transposable elements. The library was used as query to run tblastx against the *C. elegans *genome. Those hits with a percentage identity greater or equal than 90% and with an e-value less or equal than 1e^-100 ^were considered significant. Then, for each perfect duplicon detected using OrthoCluster, we looked within the duplicon and within a flanking region of 5,000 bp for associated transposons.

### Nematode Strains and Maintenance

All strains were maintained at 20°C, and all manipulations were conducted using standard methods.

### Isolation of genomic DNA

Genomic DNA were isolated from the various *C. elegans *strains using a modified single worm lysis genomic DNA preparation protocol [46]. Briefly, the worm lysis solution is composed of: 10 mM Tris (pH8.3), 50 mM KCl, 2.5 mM MgCl_2_, 4.5% Tween 20, 0.05% gelatin and 0.06 μg/μl proteinase K. For isolation of each particular strain, 100 worms were selected and placed into 30 μl of the lysis solution. The nematodes were then freeze-cracked twice and incubated at 60°C for one hour followed by one hour at 95°C to inactivate the enzyme. The resulting supernatant was used as template for subsequent PCR reactions. The F56A4 cosmid was purified via standard plasmid isolation procedures and diluted to 20 ng/μl with 1× TE for use in further steps as PCR template.

### PCR analysis of the 319 bp unique sequence

Three PCR primers–319L (aaccgattccaccgagaact), 319IR (caaccaatttccaaaatatcttca) and 319OR (ttttgctattgttgggcattc)–were designed to detect the 319 bp unique sequence (Figure 3a). The expected PCR products from reactions containing the primers 319L and 319OR are 1,319 bp (if the 319 bp unique sequence exists in the second copy) and 1,000 bp (if the 319 bp sequence is absent from the duplication unit), respectively. The expected PCR product size as a result of a reaction containing the primers 319L and 319IR is 750 bp.

### PCR analysis of the junction between the two largest duplicons

Four PCR primers–DupOL (ggtaatacttgcaccaacggt), DupOR (catacgaacatcgcggactcc), DupIR (cgatagacagacattggcaac) and DupIL (gagaaagattttggcgggaac)–were designed to amplify the leftmost boundary of the leftmost duplicon, the junction between these two copies, and the rightmost boundary of the rightmost duplicon (Figure 3a).

## Authors' contributions

NC and DLB conceived the study. IAV, AKM, JCH, MTG, and RCJ conducted the experiments NC and IAV wrote the manuscript with input from DLB and MTG. All authors have read and approved the final manuscript.

## Supplementary Material

Additional file 1 List of all 1,980 perfect segmental duplications in *C. elegans*. Click here for file

Additional file 2 **Size distribution on each chromosome of perfect duplications in *C. elegans *measured in (a) number of genes and (b) base pairs (kb)**. The y-axis represents the frequency in a logarithmic scale (base 10) of the frequency of a specific duplicon size. Thus, those bins with no visible bar mean that only one duplicon is observed for that particular value. For (b), each N value in the x-axis represents all those duplicons that fall in the range [N-1..N) kb. Click here for file

Additional file 3 **Example of imperfect duplicons that are merged from neighboring perfect duplicons by allowing some mismatches**. The clusters that have same prefixes are duplicon pairs. For example, CL-2469_1 and CL-2469_2 is one duplicon pair. The perfect segmental duplications CL-2469, CL-2470 and CL-2471 occur in the neighboring region on *Chromosome V*, whereas CL-2482 is dispersed in the upstream region of this segmental duplication (not shown). Click here for file

Additional file 4 **Twelve pairs of gene models found within the largest pair of duplicons that are not identical**. These gene models were expected to be identical because these duplicons are essentially identical in the protein coding regions at the DNA level. There is a 13^th ^pair not shown involving gene F56A4.3 (see text for details). Click here for file

Additional file 5 Revised gene models in the largest segmental duplicons. Click here for file
