# Supplementary figures and images for: Polymorphic segmental duplication in the nematode Caenorhabditis elegans
Source: BMC Genomics. 2009 Jul 21;10:329. doi: 10.1186/1471-2164-10-329 (PMC2728738; doi:10.1186/1471-2164-10-329)

a

I

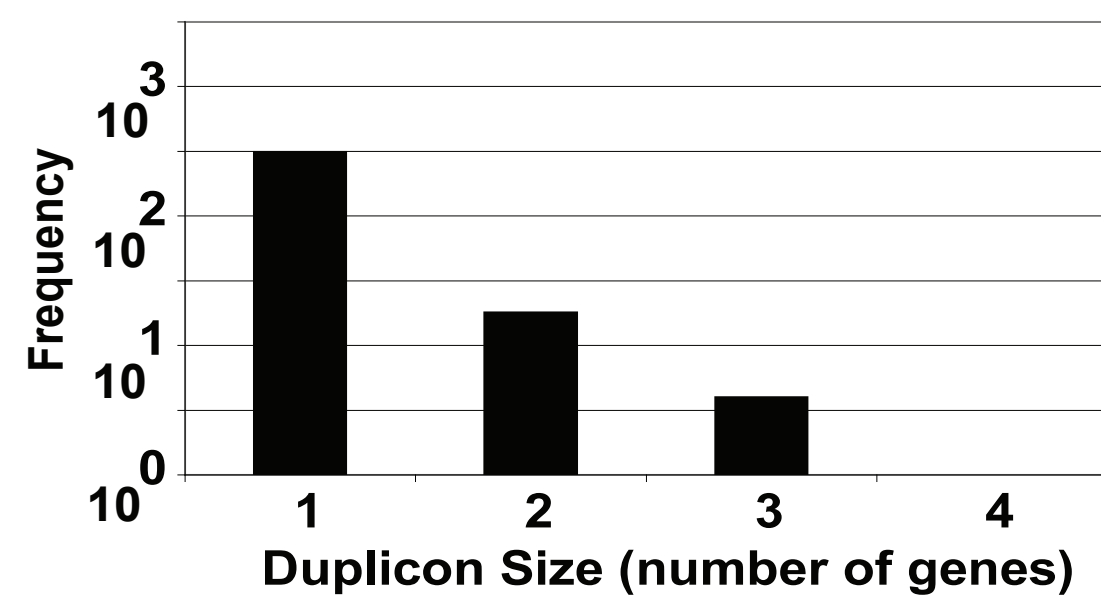

II

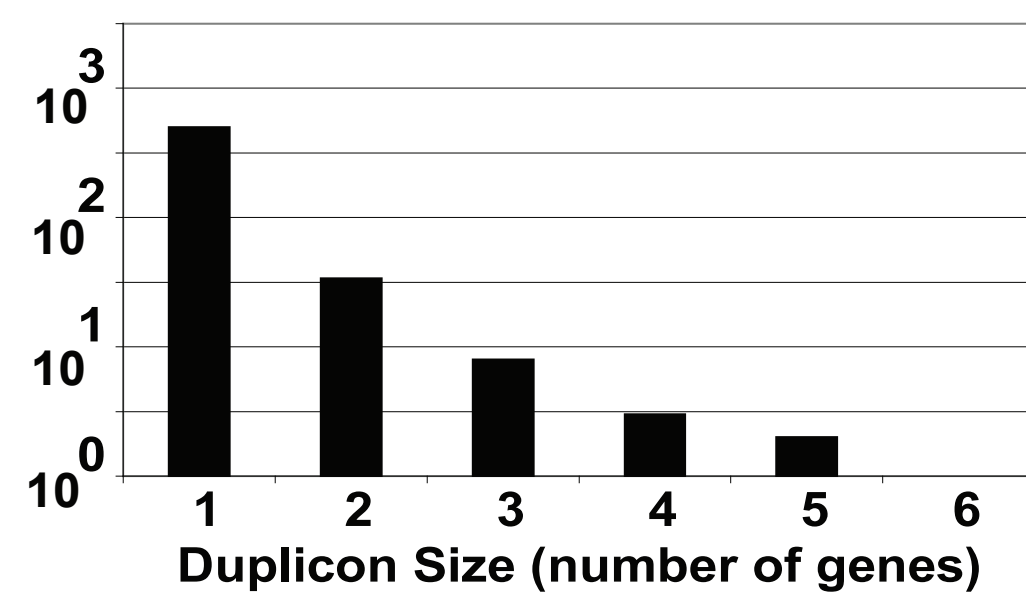

III

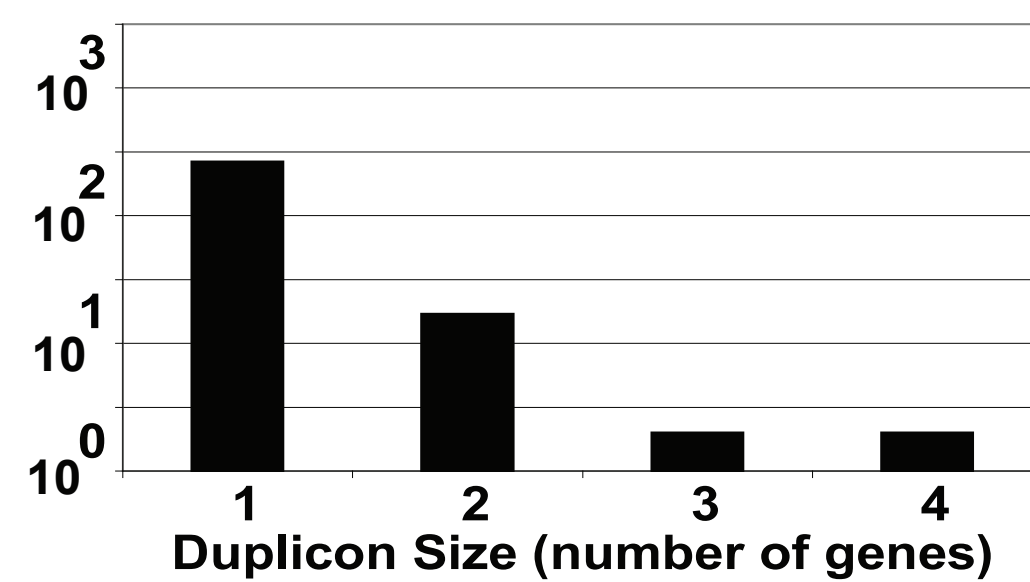

IV

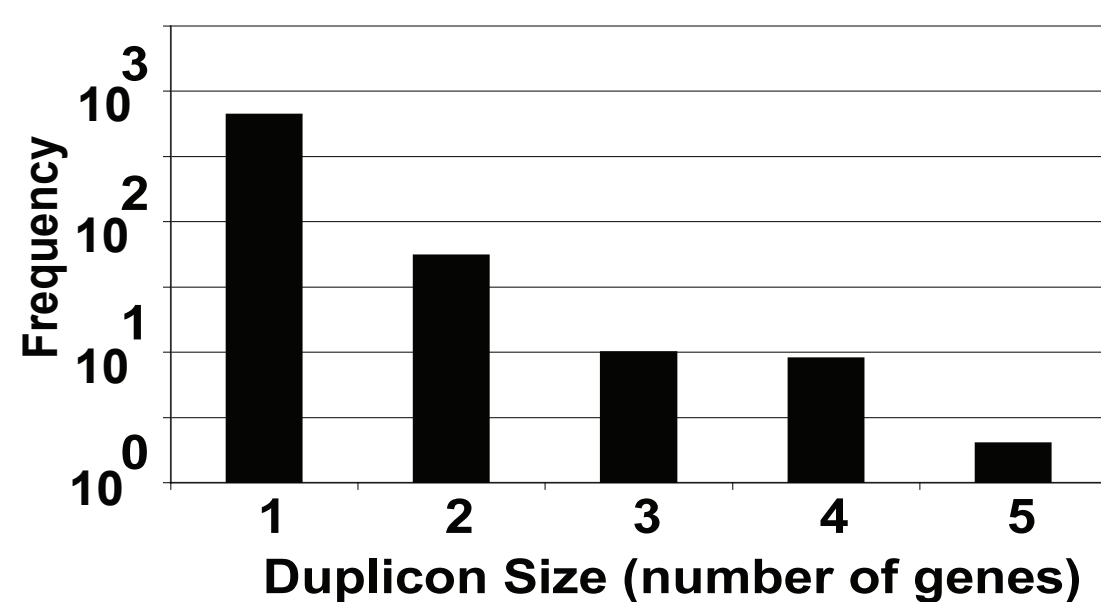

V

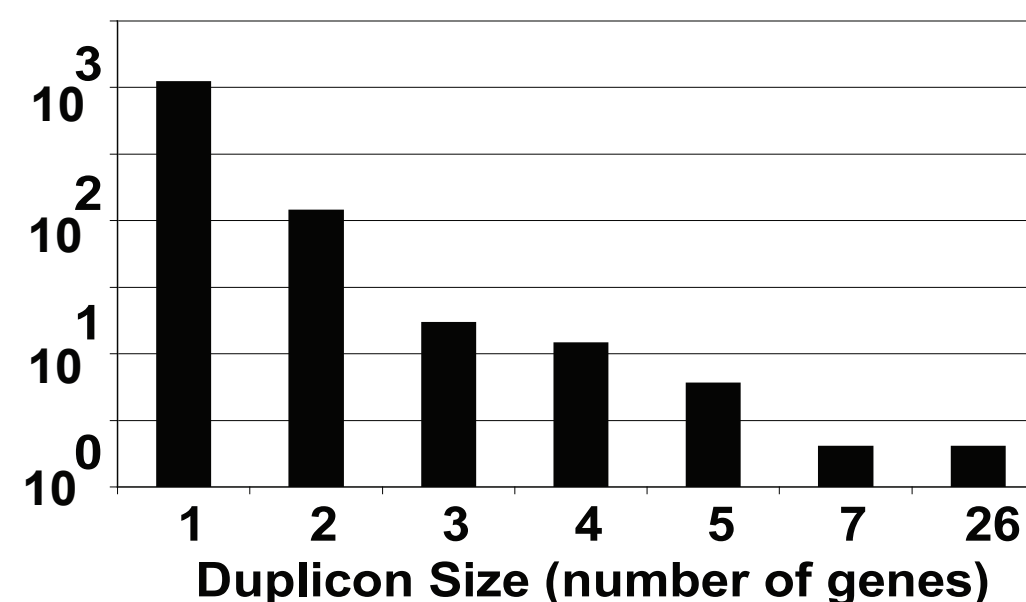

X

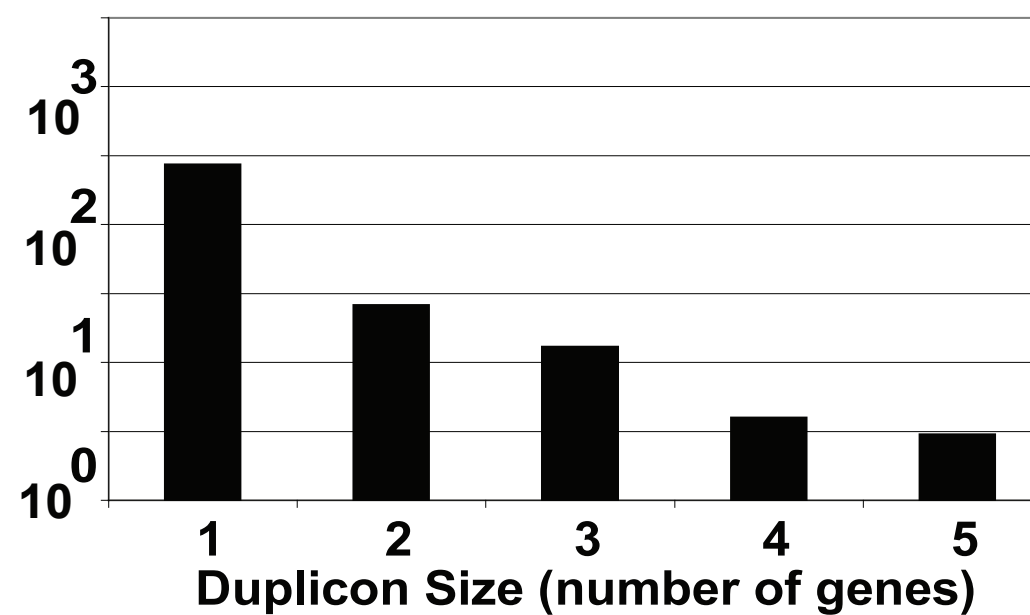

b

I

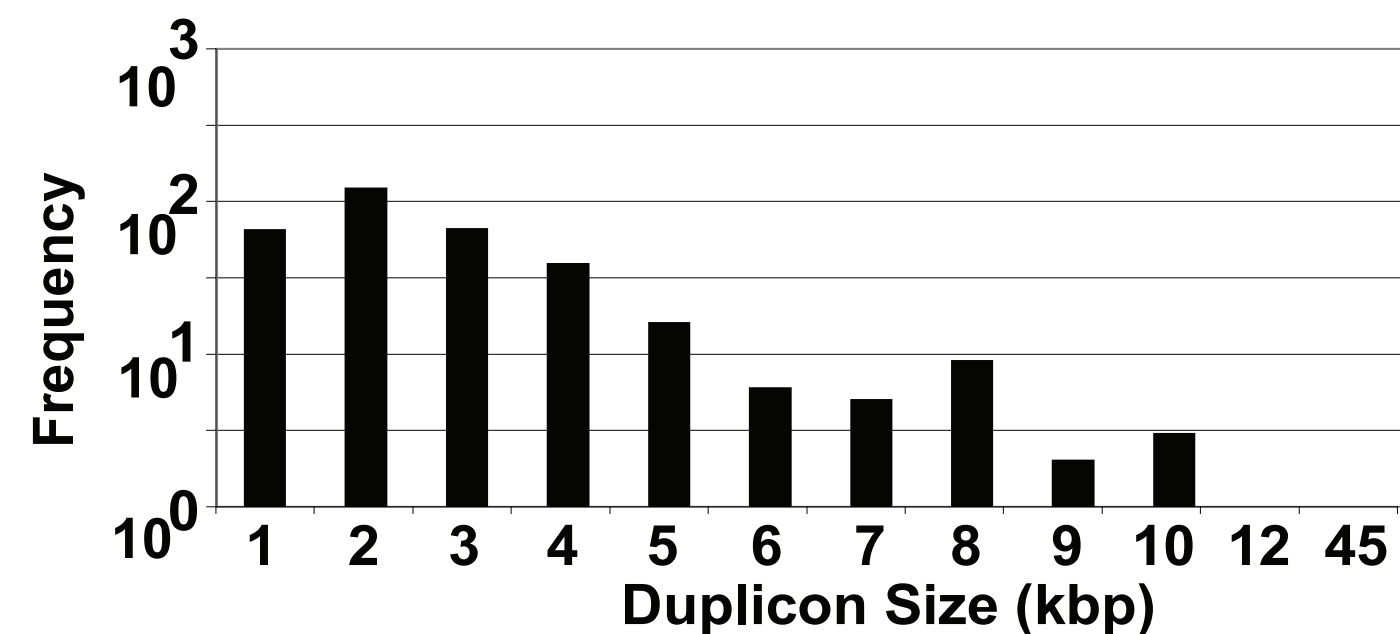

II

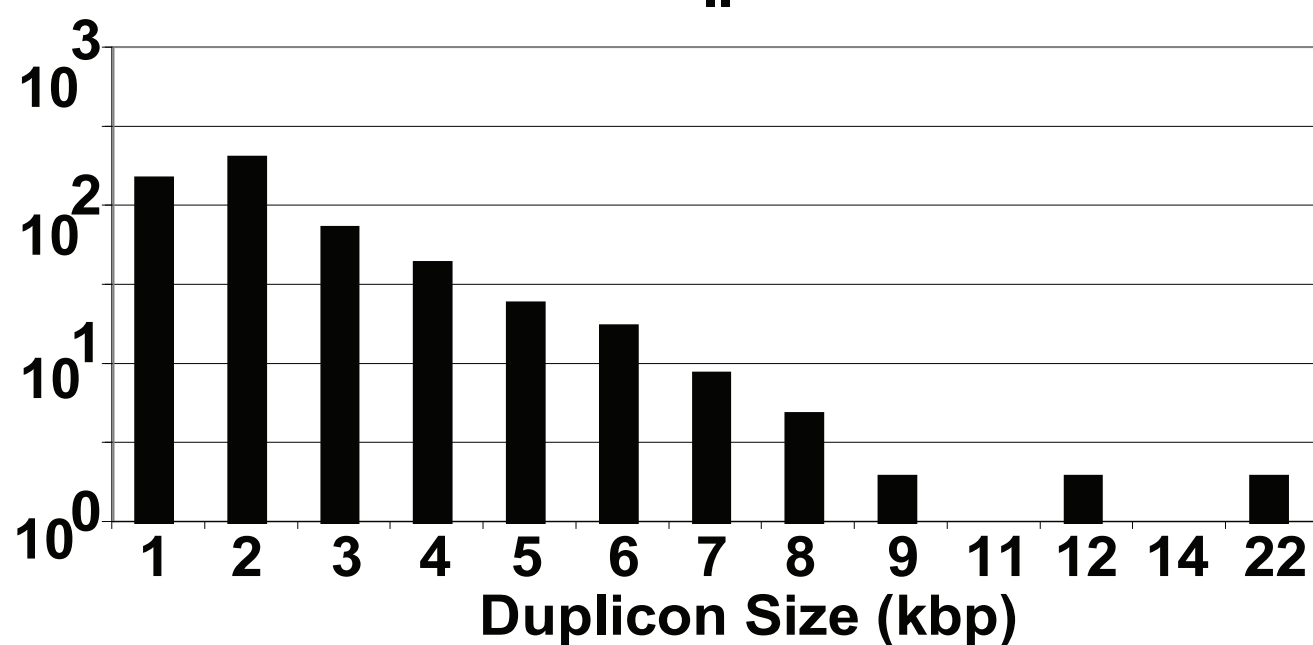

III

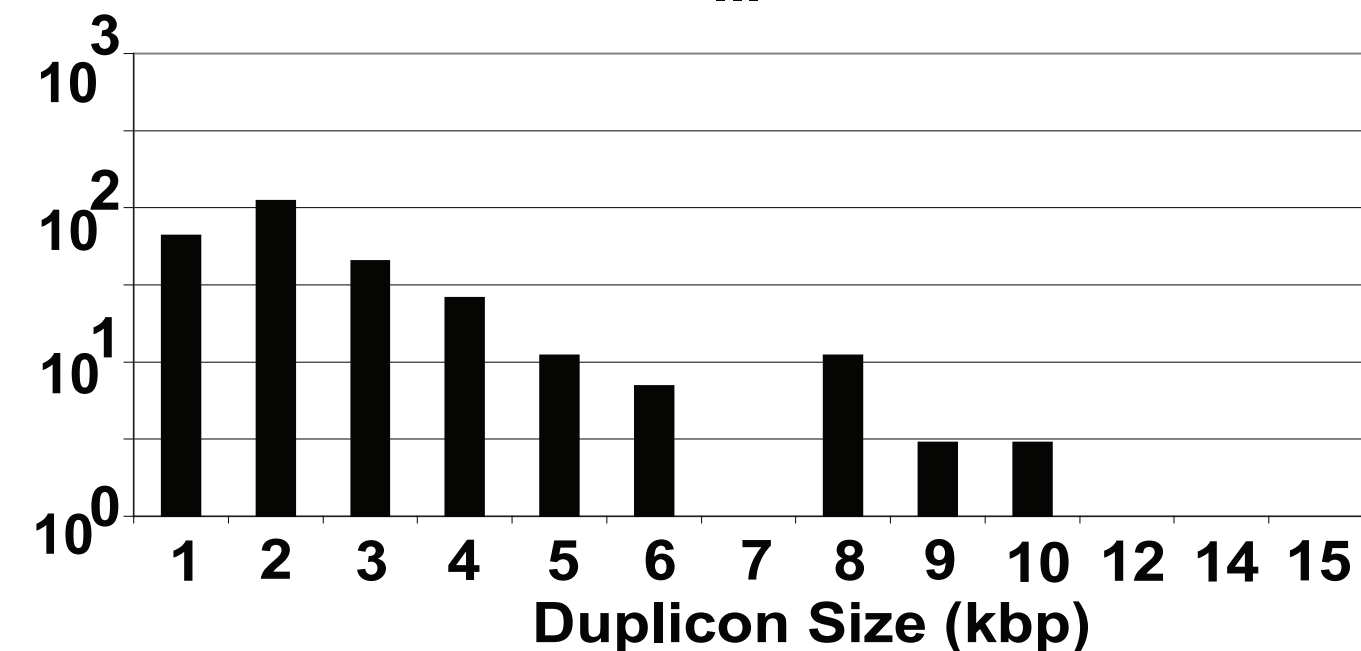

IV

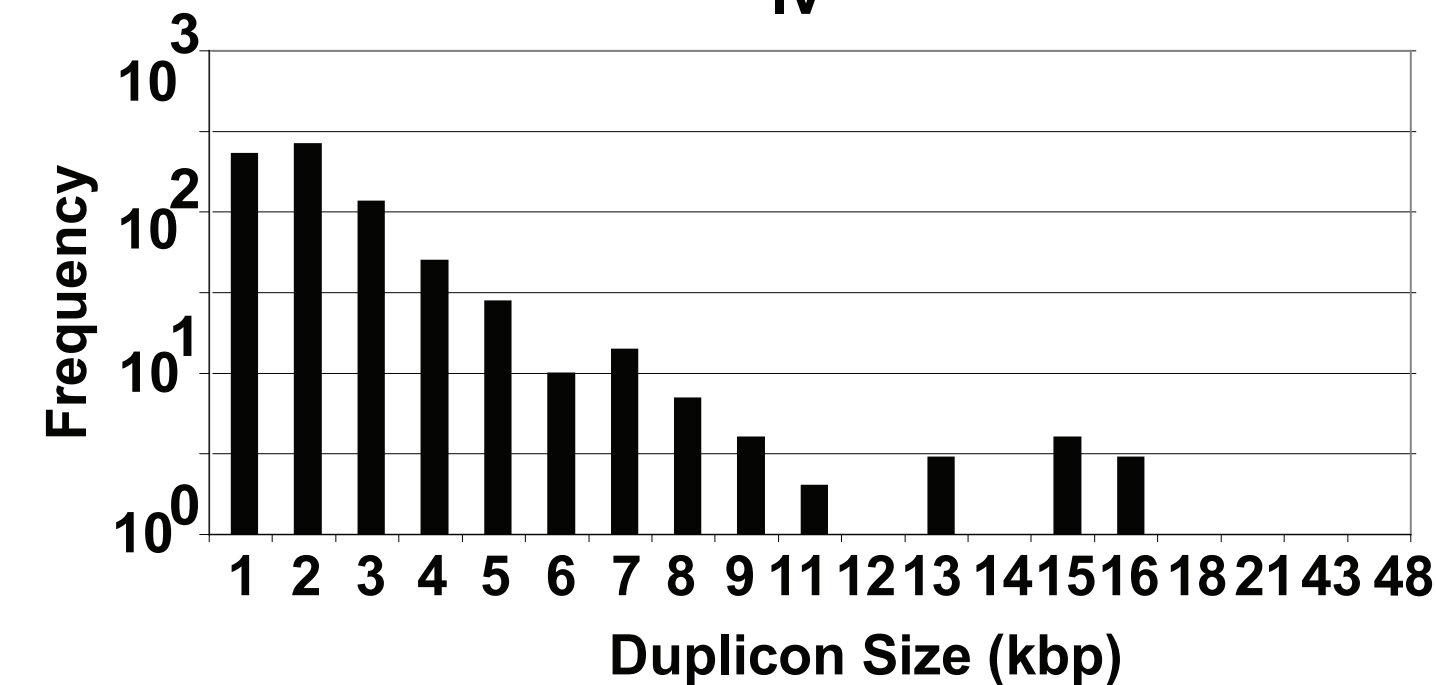

V

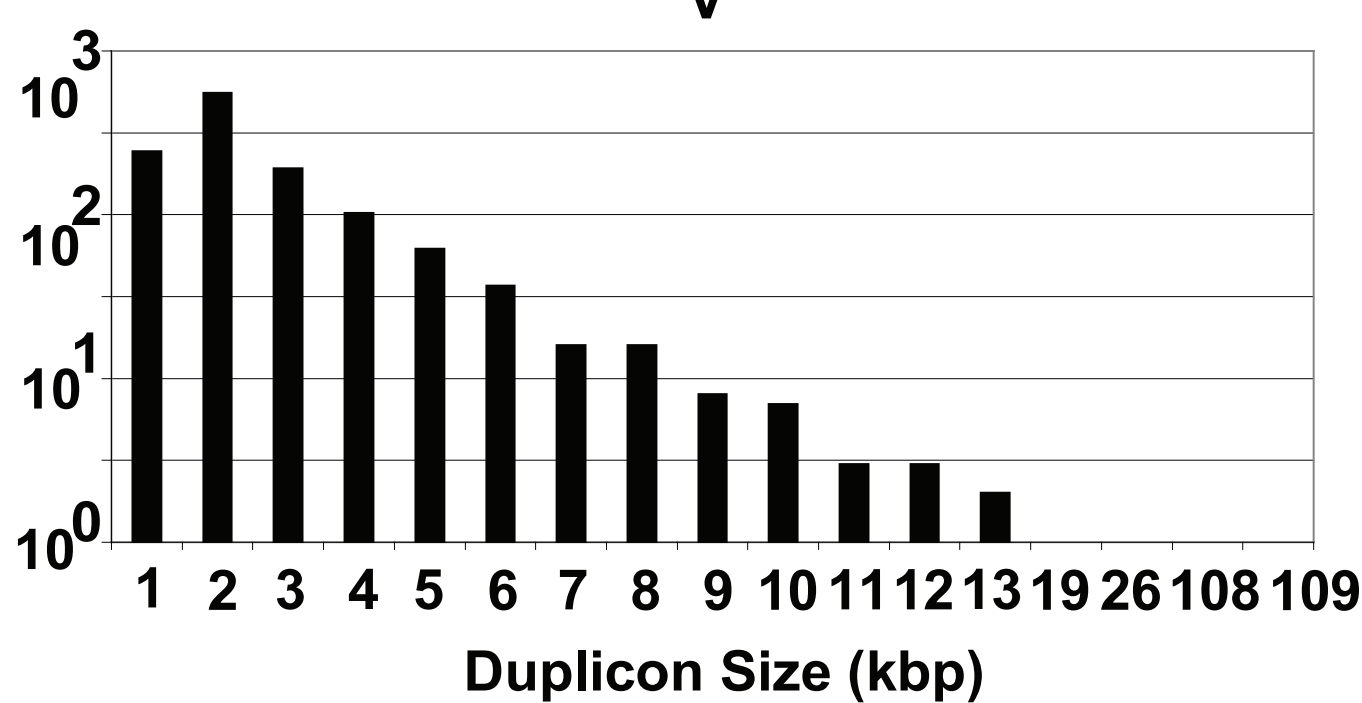

X

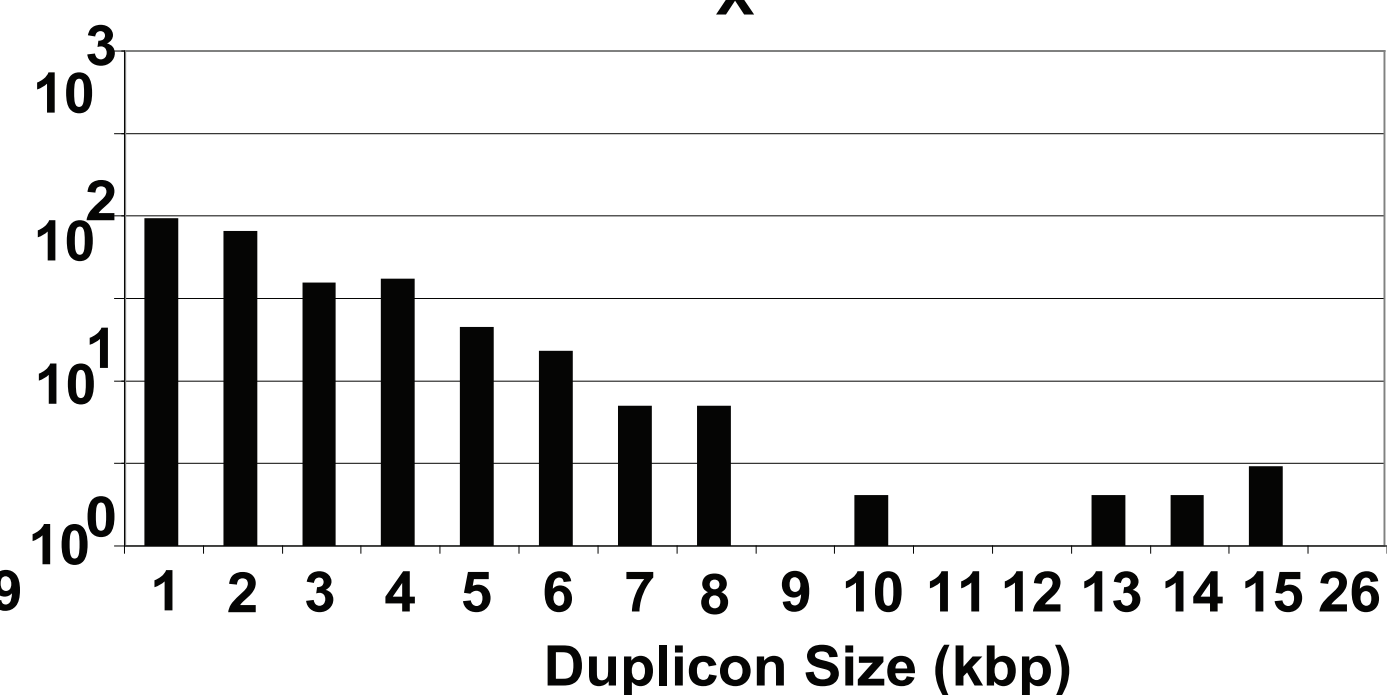

Supplement: Additional file 2 — Size distribution on each chromosome of perfect duplications in C. elegans measured in (a) number of genes and (b) base pairs (kb). The y-axis represents the frequency in a logarithmic scale (base 10) of the frequency of a specific duplicon size. Thus, those bins with no visible bar mean that only one duplicon is observed for that particular value. For (b), each N value in the x-axis represents all those duplicons that fall in the range [N-1..N) kb. [file 1471-2164-10-329-S2.pdf]

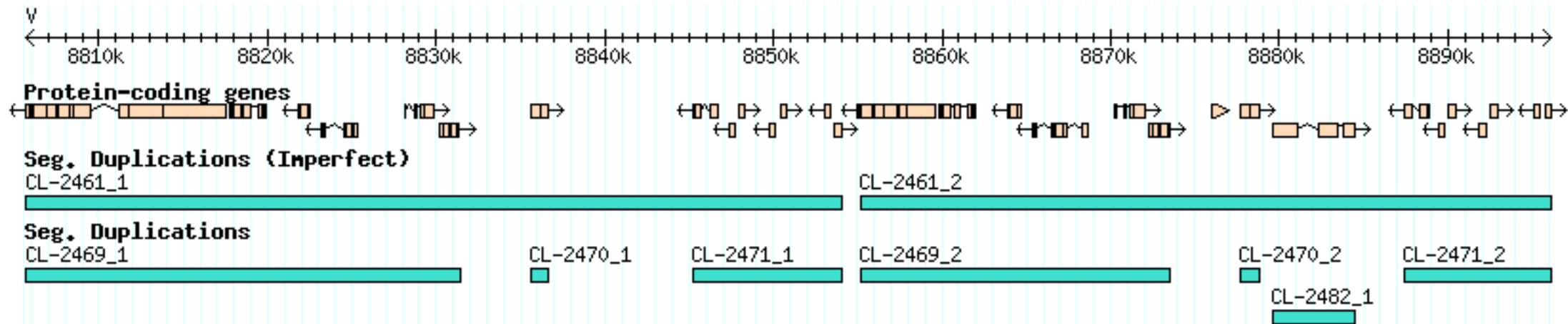

Supplement: Additional file 3 — Example of imperfect duplicons that are merged from neighboring perfect duplicons by allowing some mismatches. The clusters that have same prefixes are duplicon pairs. For example, CL-2469_1 and CL-2469_2 is one duplicon pair. The perfect segmental duplications CL-2469, CL-2470 and CL-2471 occur in the neighboring region on Chromosome V, whereas CL-2482 is dispersed in the upstream region of this segmental duplication (not shown). [file 1471-2164-10-329-S3.pdf]

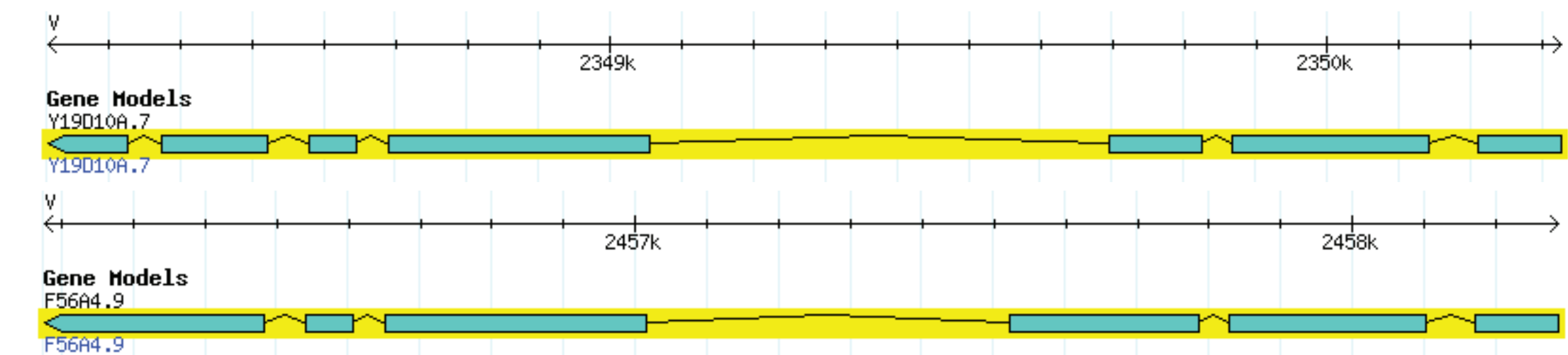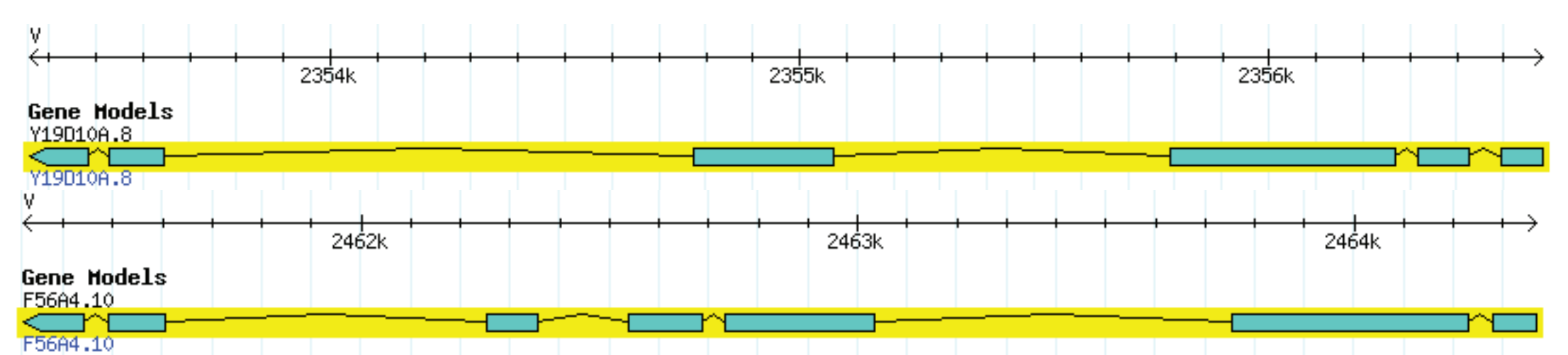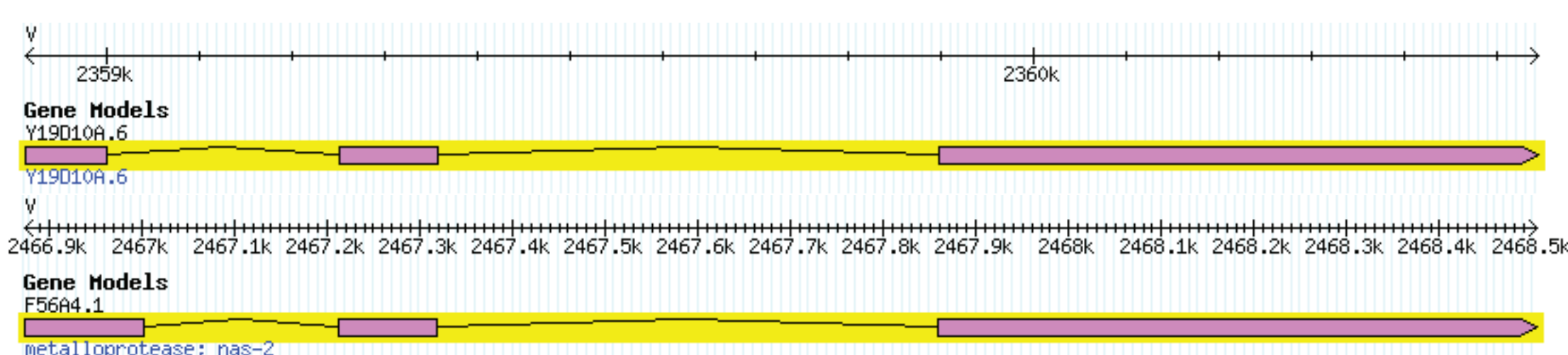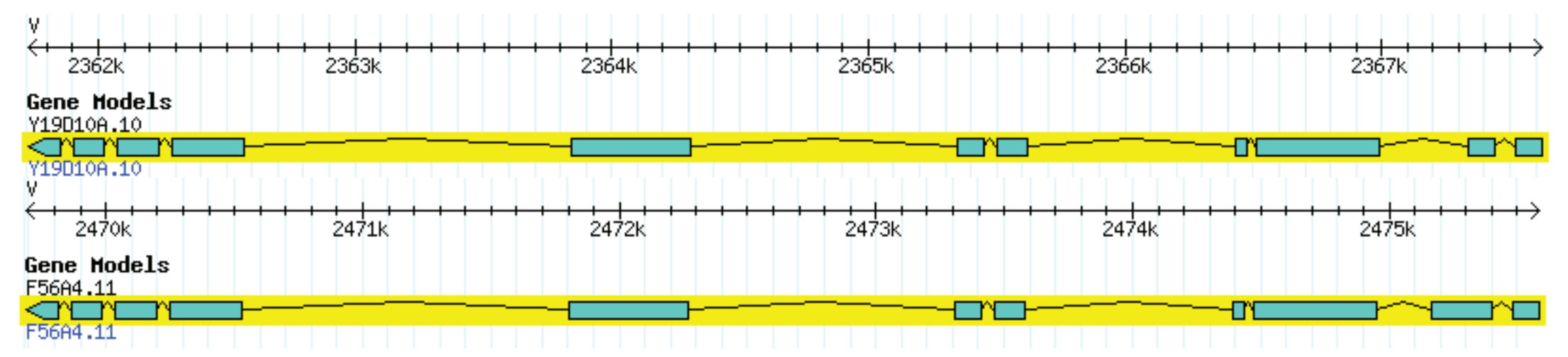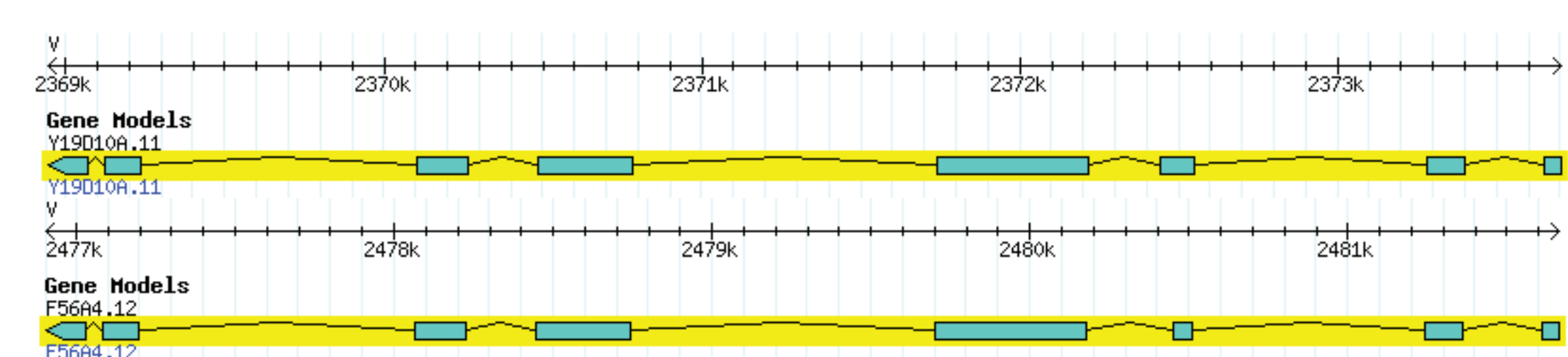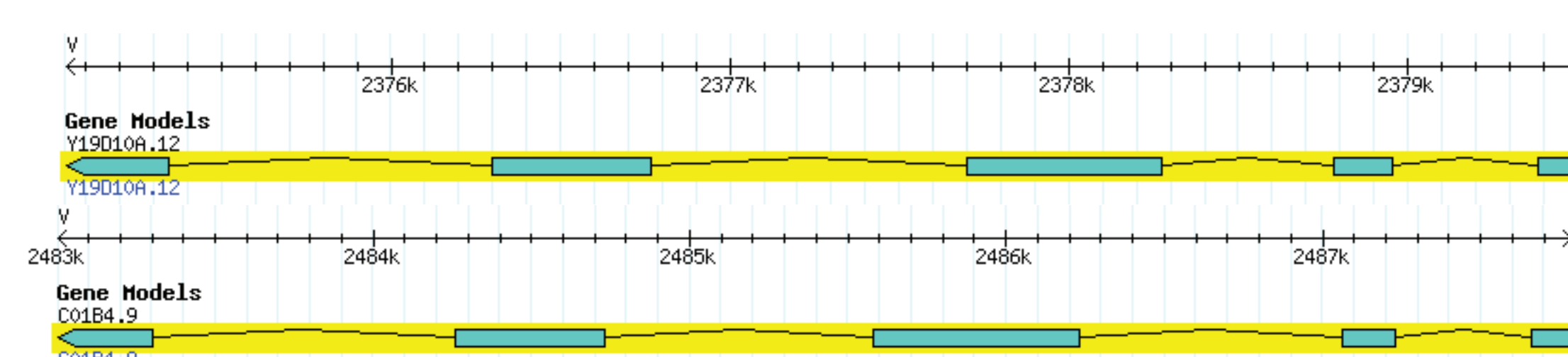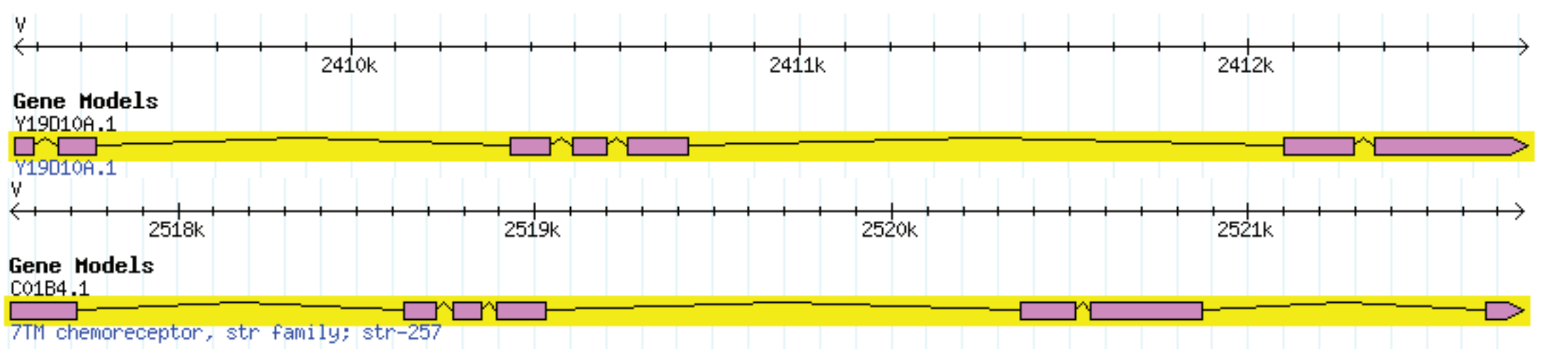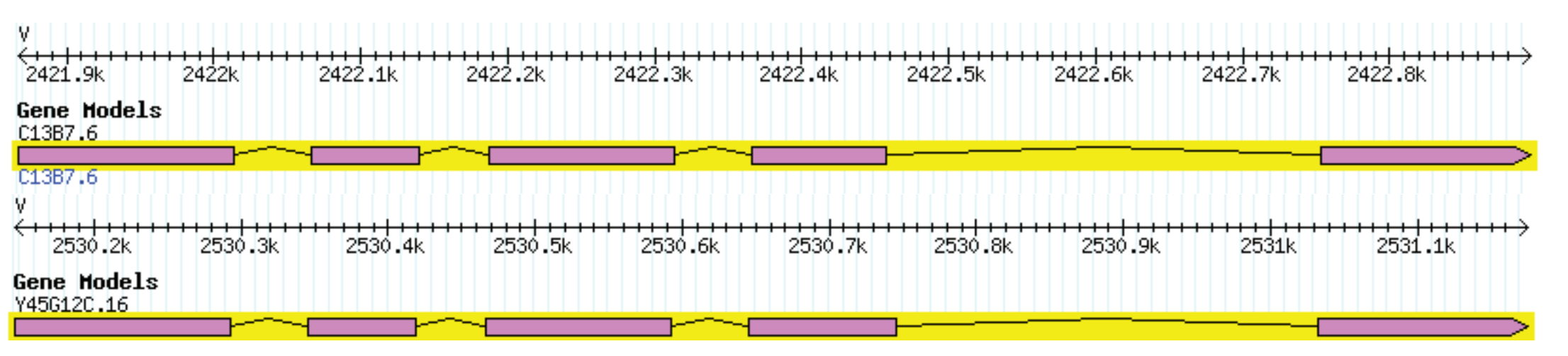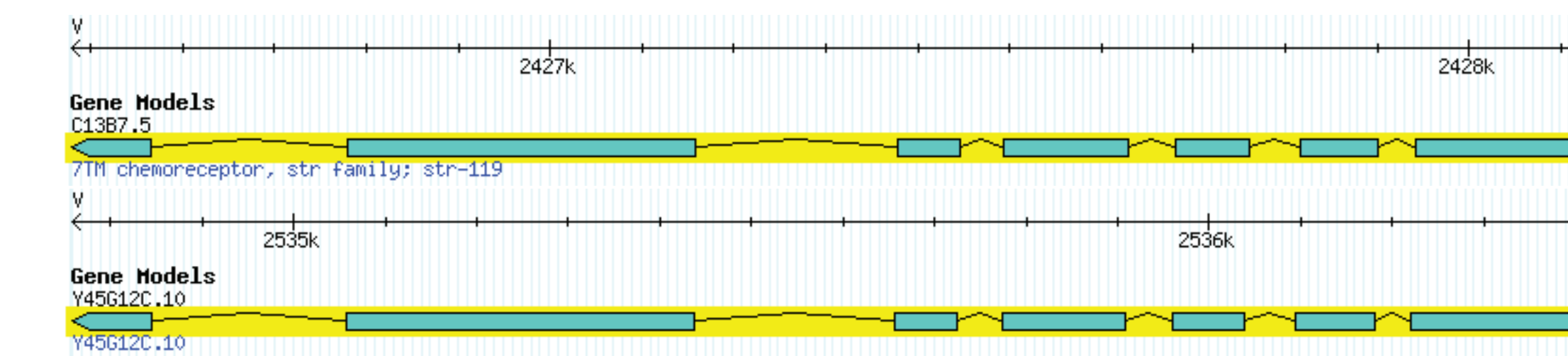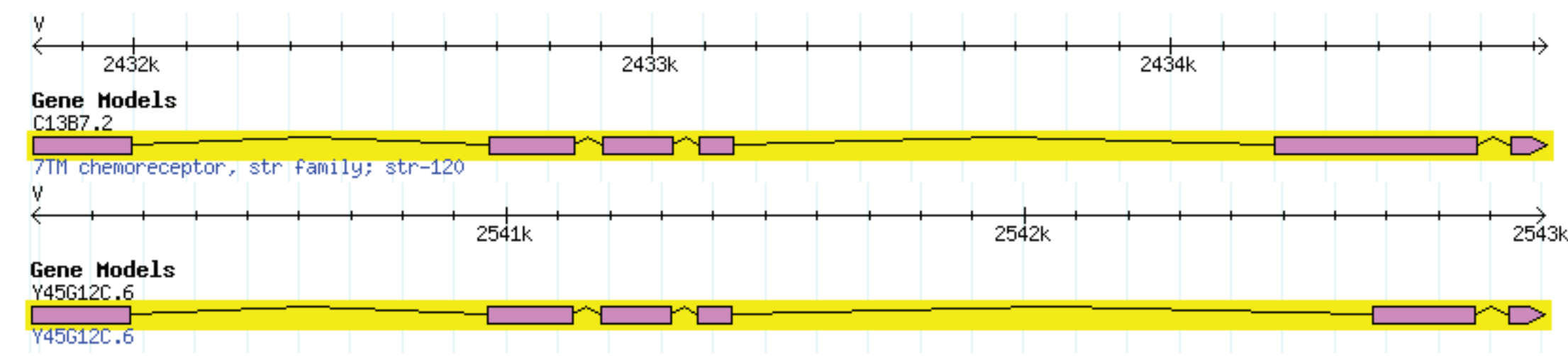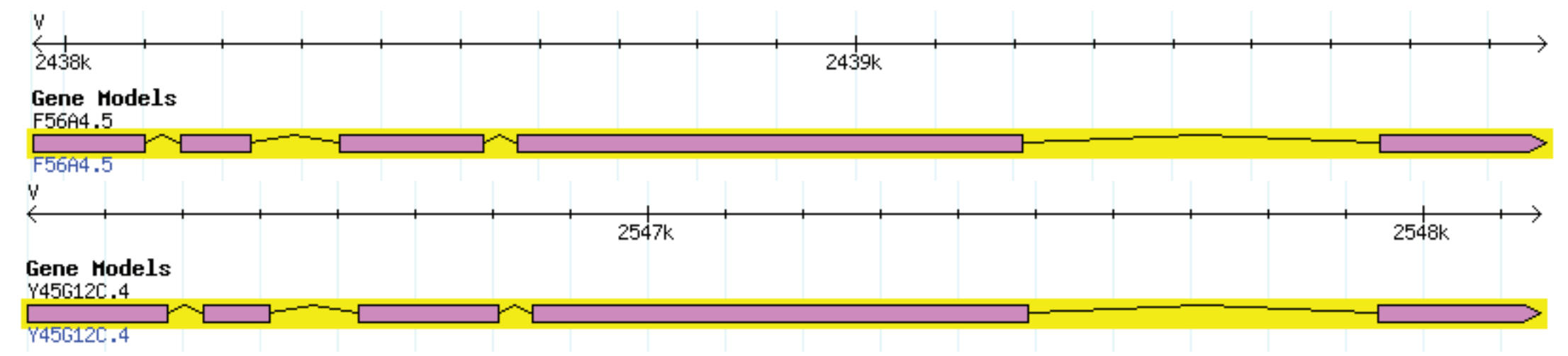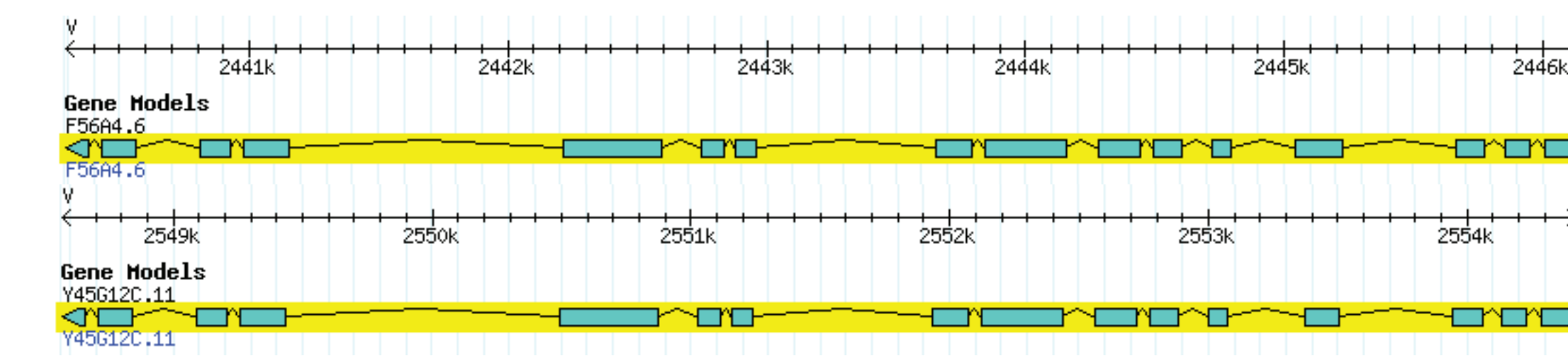

Supplement: Additional file 4 — Twelve pairs of gene models found within the largest pair of duplicons that are not identical. These gene models were expected to be identical because these duplicons are essentially identical in the protein coding regions at the DNA level. There is a 13th pair not shown involving gene F56A4.3 (see text for details). [file 1471-2164-10-329-S4.pdf]
